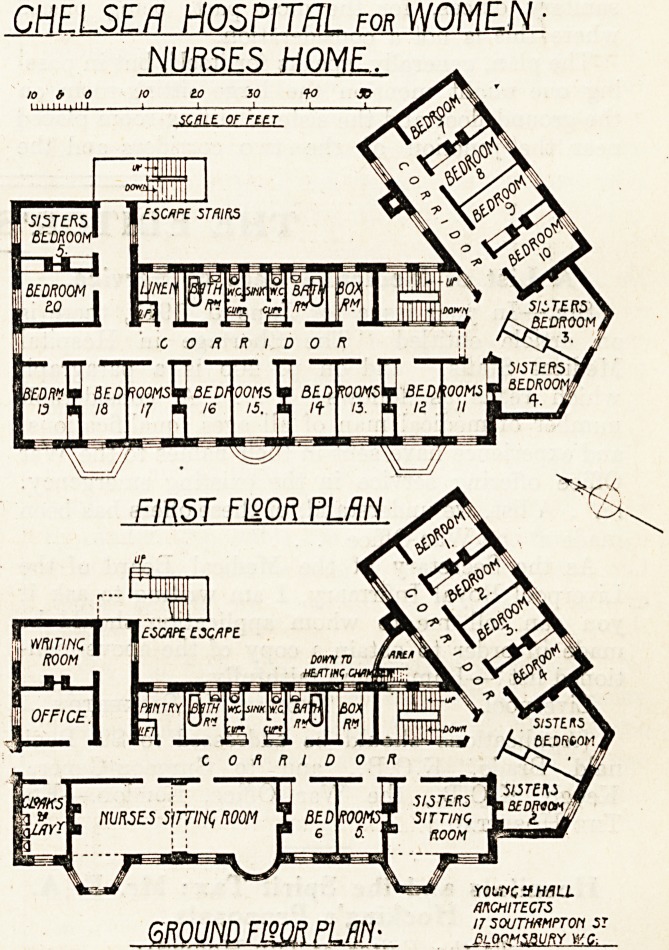# The New Chelsea Hospital for Women

**Published:** 1915-07-10

**Authors:** 


### HOSPITAL ARCHITECTURE AND CONSTRUCTION.

### The New Chelsea Hospital for Women.

THIS hospital is being rebuilt on a site in Arthur Street, Chelsea, the gift of Lord Cadogan, K.G. When completed the new building will consist of three detached blocks—namely, the main hospital, the pathological block, and the nurses' home. The main block only is in course of erection.

Our attention is naturally directed at the outset to the principal or ward floors, which are at the first and second floor levels. It will be noticed that the general arrangement of each of the two blocks or wings has been governed by the fact that a number of comparatively small wards is required. This consideration, together with the limited site, have had some restricting influence on the general planning and scheme.

The difficulties have been well met in the arrangement shown, which consists of two ward blocks, separated by a central block to which they are connected by cross-ventilated corridors. The plan as regards the ward blocks being symmetrical, a description of one will suffice.

CHELSEA HOSPITAL FOR WOMEN.

FIRST FLOOR PLAN.

The floor space per bed appears to be ample, and the lighting and ventilation of each sufficient, though here, of course, the limitations of the site have made their influence felt. Furthermore, the fact of a number of small wards having to be grouped together has necessitated economical planning in the matter of sanitary offices. The placing of these in their proper relation to each and all of the wards has been well considered, and they are equally accessible from the wards they are intended to serve. The planning of these offices marks such a departure from the usual method adopted in a hospital building that it calls for special remark, and we deal with this point later.

### North and South Wings (First Floor).

There are two wards for four beds each in the front of the building and one at the rear. A ward for six beds is also in the front. These wards are conveniently reached by a wide, straight corridor extending the whole length of the building, facilitating easy supervision and opening at its north and south ends on to wide balconies which in turn give access to external escape staircases.

The sister's room between the two four-bed wards in the front is conveniently placed, as also the linen-room in juxtaposition to it. Opposite this office is a store-room, fitted also as an examining-room with a lavatory basin.

Next this room are a store-room for patients' clothes and the ward kitchen, the latter being fitted with sink, dresser, and stove. Near the wards is a sink-room, with a w.c. for nurses leading off from it.

The south is practically the same as the north wing, except that a lift is provided which opens into its proper department on each floor. A ventilated linen shoot is also provided, and is accessible from a balcony leading off the main corridor, thus preventing atmospheric communication between the various floors.

### Central Block.

This block contains, in the front, matron's sitting-room, bedroom and bathroom, main staircase, and electric lift.

Beyond the main corridor is the operation suite, comprising anæsthetic room, theatre, sterilising and wash-up room, surgeons' room with a bathroom leading out of same, and a nurses' room. A changing-room with necessary fittings is provided. This room has a door leading to an outside staircase.

On the north side of this department is a passage leading to the theatre, for the use of visitors. The theatre has a large window and a top light on the north side. The whole of these rooms have the necessary fittings, such as sinks, lavatory basins, etc. The suite is compact and well planned.

The plan of the wings on the second floor is similar to that of the first floor, therefore no plan of this floor is given. In the central block the space over the matron's quarters is occupied by the linen store and mending-room.

### Out-Patient Department.

This has necessarily been placed on the lowest floor and occupies the basement of the north wing, access for patients being by means of an outside staircase.

The yard of entrance is covered by a glass roof to afford shelter to patients waiting.

The waiting-hall is about 56 feet by 22 feet, and has a central entrance. Leading off this is a consulting-room about 24 feet by 22 feet. Four ful for the admission of stores; the two store-rooms shown opening from the area next the dispensary should give ample accommodation for the storage of drugs in large quantities.

A small space has been enclosed in the corner of the waiting-hall for the use of the almoner.

The out-patient department can be entered from the hospital corridor, but its severance from the hospital atmosphere, as far as is possible in a plan of this kind, is secured by a cross-ventilated lobby.

BASEMENT PLAN.

GROUND FLOOR PLAN.

examining cubicles are provided, all having windows on the north side. In connection with these rooms are eight dressing-rooms placed behind them. An exit passage is arranged from the consulting-room giving access to the medicine waiting-room and exit, and also to the waiting-hall.

The customary arrangement of seats in the medicine waiting-room is shown, ensuring patients receiving their medicine in their proper order.

The dispensary occupies the remaining space in this wing, and has a door opening into an area, use-

In the front area are two water-closets for the patients, which can be entered from the open air.

The space available for this department, although restricted, has been made good use of.

### Administrative Features.

The remaining space on the basement floor is occupied by a central block containing porters' bed-rooms and bathroom, main staircase and lift, sterilising-room, pump-room, boiler-house and coal-stores, and general sanitary accommodation.

the south wing are stores and receiving-room, soiled-linen room, Jews' kitchen, larder and scullery, and porters' sitting-room. The unallotted space will probably be arranged for electrical work.

On the ground floor the north wing contains the secretarial department, board-room, staff-room, cloak-room, waiting-room, and quarters for resident medical officers, with their proper complement of sanitary offices. The south wing contains kitchen offices, nurses' dining-room, servants' hall, stores and pantries, and nurses' cloak-room; whilst in the central block are the entrance-hall and waiting-room, porters' office, telephone-room, lift, and staircase.

At the rear beyond the main corridor are the matron's office, four single-bed wards, operation-room, bathroom, and ward kitchen for septic cases.

The pathological block consists of four rooms—viz., *post-mortem* room, museum, and laboratory, enterable from an open lobby, and a small mortuary connecting with the *post-mortem* room, and having a door for access from the yard. At the end of this block is a shed for cars.

### Tradition Abandoned in the Sanitary Offices.

Everyone familiar with hospital planning for the last, say, fifty years in this country cannot have failed to notice the manner in which these offices have been rigorously placed outside the ward, from which they could only be reached by a cross-ventilated lobby, or in some instances an open bridge, the obvious object being absolutely to prevent any passing of air between the sanitary offices and the ward. This practice has been so universal that to any not familiar with the working of a hospital and the excellence of the fitments of the sanitary offices and plumbing it must come as something of a shock to find the universal practice completely abandoned as is the case in the new buildings of the Chelsea Hospital for Women. The justification for this procedure urged by one of the authors of the design, Mr. Keith D. Young, F.R.I.B.A., seems to be that the sanitary offices are not entered directly from any ward, but from the corridor. Apart from this he does not consider that the cut-off lobby is the absolute necessity it was formerly thought to be, for he claims that as, in use, the lobby doors are invariably fixed open and the windows almost always shut, the efficacy of the plan vanishes.

Further, in a letter written to Mr. William Milburn by Mr. Keith Young, which was read before the Royal Institute of British Architects on February 17, and published in the *Journal of the Royal Institute of British Architects* of March 8, 1913, he says:—

I have gradually been coming to the conclusion that the disconnecting lobby is not a necessity. In the old days, when sanitary plumbing was a lost art, or, rather, had not been developed, and fittings, and connections, and everything else were all of the crudest description, it no doubt was necessary to interpose a ventilated lobby between the sanitary offices and the ward; but now, with the almost perfection that sanitary work has got to, I see no real necessity for it. Moreover, you will find that wherever the lobby is provided the nurses invariably fix the doors open, and so render it of no effect. The practice has never been adopted in Continental hospitals to any extent, while in American hospitals, so far as I know, they depend very much upon their ventilation arrangements to prevent air passing from the sanitary offices to the wards. This, personally, I do not believe in a bit. I would not, of course, enter the sanitary offices direct from the wards, but it would be an immense help in planning if we could get rid of the projecting towers. Another point that I should like to make, although no doubt it has occurred to you, and that is putting the bathrooms out in a projecting sanitary tower with a cross-ventilated lobby is nothing else than a blunder. A bathroom is not, of course, a sanitary office in the same way that a water-closet or a sink-room is, and the ventilated lobby may be a positive danger to a patient passing through it after having had his bath. In children's wards or children's hospitals I always arrange, if possible, that the bathroom shall be entered direct from the ward.

How many architects are there who share these conclusions? How many, again, declare they have often felt from personal experience that the danger, especially in winter, of exposing patients who were just allowed to get up from their beds to the draughts which they were subjected to in passing through these lobbies was one which should be removed at almost all costs.

On these points we invite discussion.

We may add that in a sketch supplied by Mr. Young the lobby to the w.c.s is shown ventilated into the open-air above the ceiling of the w.c.s, which is purposely kept for that purpose at a lower level than the ceiling of the lobby.

Nurses' Home.

Two floors only of this building are illustrated—viz., the ground- and first-floor plans.

The problem in planning a nurses' home is simply to design a healthy dwelling. This object has been well attained by providing a separate bedroom for each nurse, each of which has a sunny aspect at some part of the day, the necessary sanitary offices, for the most part, being placed where this is not a consideration.

The plan, generally, speaks for itself, but in passing one might mention the large sitting-room on the ground floor and the sisters' sitting-room placed near the junction of the two corridors and the main staircase, and the sisters' bedrooms similarly placed on both floors. A cloak-room and lavatory is placed near the entrance, and an office and a writing-room at the rear of same, and well away from the main corridor. A box-room is provided on each floor, and a pantry on the ground floor, and a linen-room over the first floor. A lift communicates with the different floors. An escape staircase is shown in a position where it would be useful.

Generally speaking, the plan is a good one, and we consider the building should fulfil its excellent purpose.

The architects are Messrs. Young and Hall, and the engineering work (hot-water and steam electric work and lifts) is under the charge of Mr. C. T. Walrond, C.E.

## Figures and Tables

**Figure f1:**
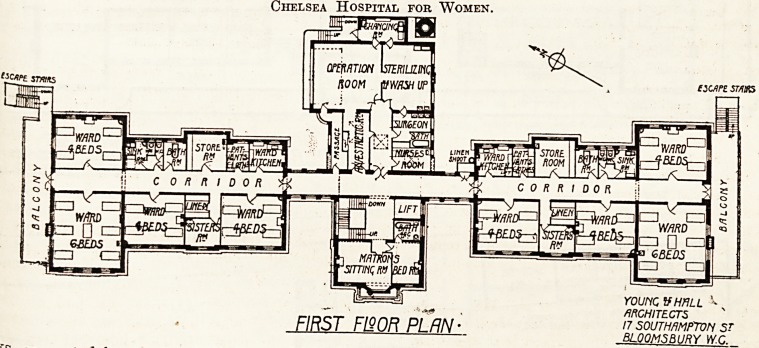


**Figure f2:**
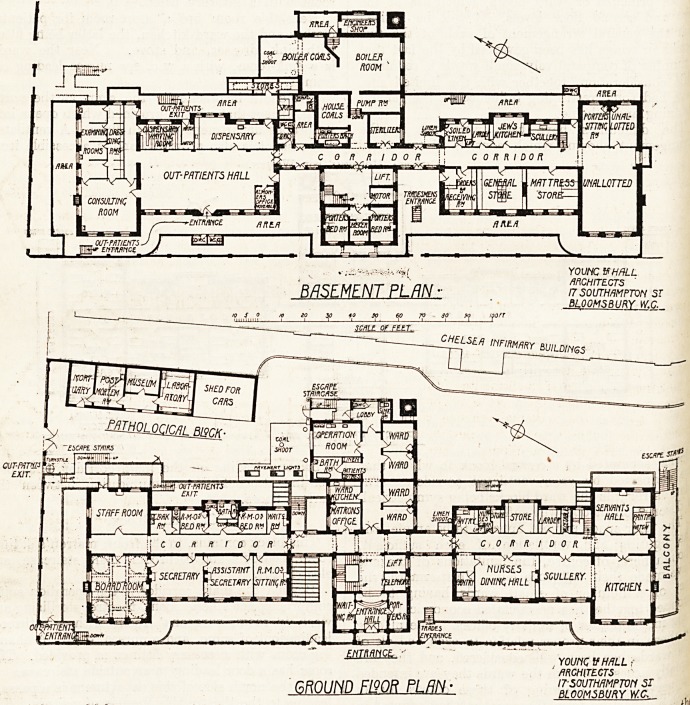


**Figure f3:**